# Facile Preparation of Granular Copper Films as Cathode for Enhanced Electrochemical Degradation of Methyl Orange

**DOI:** 10.3390/ma14112697

**Published:** 2021-05-21

**Authors:** Jiajie Xing, Min Song, Mengyao Yang, Xu Tan, Fenglin Li, Xixin Wang, Jianling Zhao

**Affiliations:** School of Materials Science and Engineering, Hebei University of Technology, Tianjin 300130, China; 17302207961@163.com (J.X.); sm950814@163.com (M.S.); Y15675306733@163.com (M.Y.); tanxudeyouxiang@163.com (X.T.); 18340355163@163.com (F.L.); xixinwang@126.com (X.W.)

**Keywords:** granular copper films, electrodeposition, electrochemical degradation, methyl orange

## Abstract

In this paper, granular copper films (GCFs) were prepared through electrodeposition in CuSO_4_ solution containing triethanolamine, and the films were used as electro-Fenton-like cathodes for degradation of methyl orange (MO). The effects of triethanolamine concentration, pH value, current intensity and temperature on the morphology of the films, as well as the MO decolorization ratio (DR), were investigated in detail. Results show that when the concentration of triethanolamine is 0.2 wt%, the prepared GCF exhibits the best performance. Under room temperature and neutral conditions, no external O_2_ or catalyst, MO is completely decolorized after 240 min. Compared with the commonly used carbon cathode, the GCF cathode can increase the MO decolorization rate by approximately 70.9%. The kinetics of the electrochemical degradation reaction is also discussed.

## 1. Introduction

With the rapid development of today’s economy and society, it is inevitable to produce a large amount of wastewater containing dye and other organic pollutants in industrial production and social life, both of which will cause great harm to the human body and the environment. There are many ways to deal with organic pollutants [1], for instance, coagulation [2], membrane treatment [3], advanced oxidation process [4], biological treatment [5] and adsorption [6]. Among them, advanced oxidation has become a hot spot in the field of water treatment due to such advantages as high efficiency, fast reaction rate and thorough degradation in the treatment of organic pollutants [7]. As one of the advanced oxidation techniques, electrochemical degradation can be achieved by heterogeneous reaction between the electrode and the organic matter, or by electrolyzing H_2_O to generate strong oxidizing substances, such as hydroxyl radical (•OH), to degrade organic pollutants in water. Compared with other methods, the electrochemical degradation method has the benefit of being a simple and clean process, with mild reaction conditions and no need for external oxidant. The efficiency of electrochemical degradation is largely determined by both the anode and cathode materials. The reported anode materials mainly include Pt [8], oxides of Ti [9], Sb [10], Sn [11], Ru [12] and Ir [13], as well as carbon-based materials [14]. The most commonly used cathode material is carbon [15,16,17,18,19]. During the electrochemical degradation process, at the cathode, the reaction of Equation (1) occurs to form H_2_O_2_ [20], which can constitute the Fenton process with externally added Fe^2+^ catalyst (Equation (2)), and the generated •OH enables the non-selective removal of various organic pollutants.

2H^+^ + O_2_ +2e^−^ → H_2_O_2_, (1)

Fe^2+^ + H_2_O_2_ → Fe^3+^ + •OH + OH^−^. (2)

In order to increase the generation of H_2_O_2_ and •OH, and accelerate the decolorization rate, such measures as blowing air or oxygen into the cathode region are generally adopted, which will raise the complexity and operating cost of the process. Moreover, there is also a problem of separation and secondary pollution of the added Fe^2+^ catalyst. Therefore, investigations on the new, effective cathode materials to accelerate the conversion of H_2_O_2_ into •OH, without adding Fe^2+^ catalyst into the system and enlarging the traditional pH range of the Fenton reaction (pH ≤ 3) [21], are of great importance. It has been confirmed that Cu and CuO_X_ could act as Fenton-like catalysts and facilitate the decomposition of H_2_O_2_ to form •OH [22,23,24]. In addition, Cu could promote the formation of H_2_O_2_ during the electrolysis of water [25]. Thus, in this paper, granular copper films (GCFs) were prepared by electrodeposition and, for the first time, were used as an electro-Fenton-like cathode for electrochemical degradation of methyl orange (MO). Compared with the commonly used carbon cathode, the GCF cathode can increase the MO decolorization rate by approximately 70.9%. The significantly improved decolorization rate of MO under neutral conditions indicates that GCFs are promising cathode materials for electro-Fenton-like systems.

## 2. Materials and Methods

### 2.1. Preparation of Granular Copper Films

A copper sheet (Cu, 2 cm × 4 cm, purity of 99.4%) was used as a cathode, and it was ultrasonically washed with ethanol and deionized water before use. A platinum sheet was used as an anode, and the distance between the anode and cathode was 2.0 cm. A measured amount of triethanolamine was added to 100 g of 0.2 M CuSO_4_ solution, and the mixture was used as the electrodeposition solution. The electrodeposition experiment was carried out under a constant current condition of 40 mA/cm^2^ at 25 °C for 2 min. After that, the copper sheet was taken out, washed with deionized water and air-dried to obtain the granular copper film (GCF) sample. When the added amount of triethanolamine was 0, 0.2 and 0.4 g, the samples were prepared and designated as GCF-0, GCF-0.2 and GCF-0.4, respectively. The morphology and crystal structure of the films were examined by a scanning electron microscope (SEM Quanta 450 FEG, FEI, Hillsboro, OR, USA), X-ray diffractometer (XRD Bruker D8 Discover, Bruker AXS, Karlsruhe, Germany) and X-ray photoelectron spectrometer (XPS ESCALAB-250Xi, Thermo Fisher Scientific, Waltham, MA, USA), respectively.

### 2.2. Electrochemical Degradation of MO

The electrolyte was 50 g of 20 mg/L MO solution containing 0.5 g of Na_2_SO_4_. The sample was used as the cathode, a platinum sheet as the anode and the distance between the anode and cathode was 2.0 cm. The electrochemical degradation of MO was carried out under a constant current condition of 20 mA (4 mA/cm^2^) at 25 °C for 60 min. After the experiment, the absorbance of the solution was measured by an ultraviolet-visible spectrophotometer (U-3900 H, Hitachi, Tokyo, Japan), and the decolorization ratio (DR) was calculated according to the absorbance of the MO solution before and after the reaction [26]. Each condition was tested three times, and the DR value was averaged.

The reagents used in the experiment were of analytical purity, and the direct current was provided by the program-controlled power supply (DHD 150 V/1.5 A, Beijing Dahua, Beijing, China).

## 3. Results and Discussion

### 3.1. Morphologies and Structures

**Figure 1:**
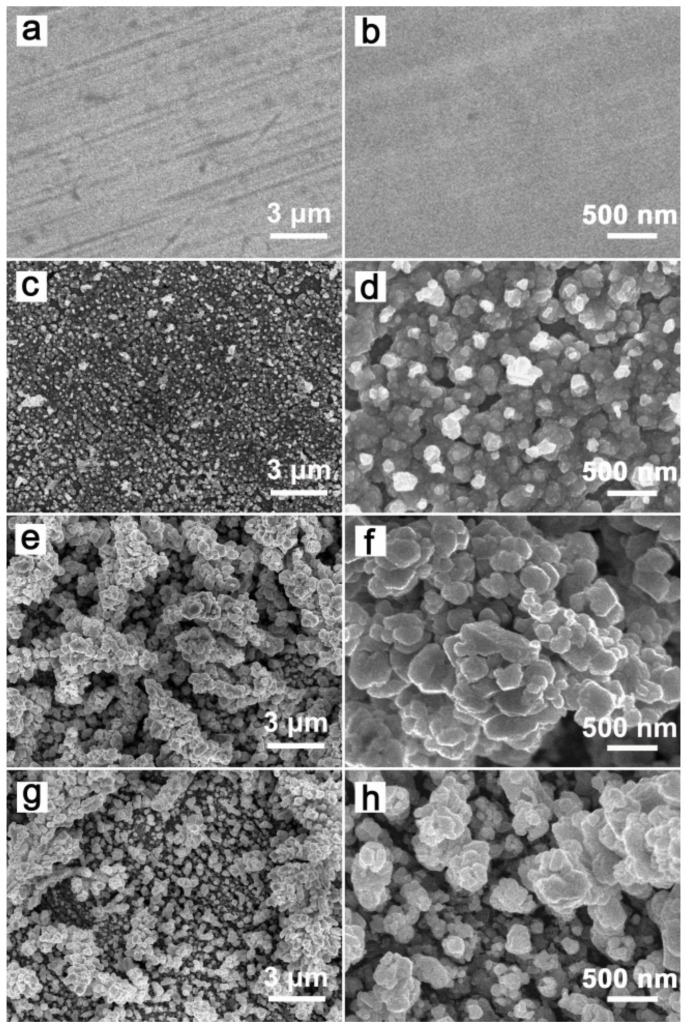
SEM images of the samples: (**a**,**b**) copper sheet; (**c**,**d**) GCF-0; (**e**,**f**) GCF-0.2; (**g**,**h**) GCF-0.4.

Figure 1 shows the SEM images of the original Cu sheet and different GCF samples. The original Cu sheet had a smooth surface (Figure 1a,b), and the surface of GCF-0 appeared rough and uneven with tightly packed particles (Figure 1c,d). The surface particles of GCF-0.2 had a loosely packed, grape-bunch structure, and the deposition layer had a large number of pores (Figure 1e). The average particle size was approximately 250 nm, and there was a large number of corners on the particle surface (Figure 1f). The surface of the GCF-0.4 also had a grape-bunch structure, which was sparse, and the number of particles was significantly lower than that of the GCF-0.2, as the particle size decreased to approximately 180 nm (Figure 1g,h). The film thicknesses of GCF-0, GCF-0.2 and GCF-0.4 were approximately 1.5, 1.7 and 1.2 μm, respectively.

**Figure 2:**
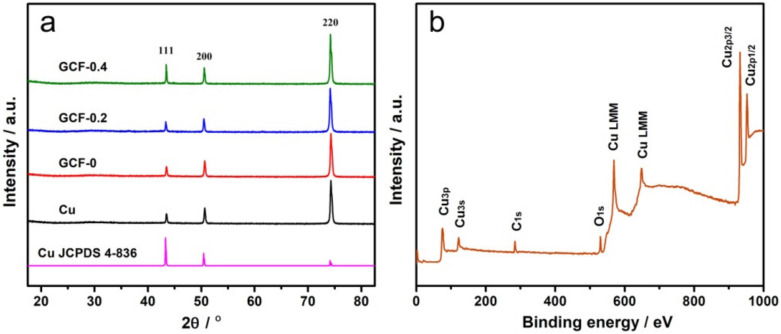
XRD patterns of the samples (**a**) and XPS spectra of sample GCF-0.2 (**b**).

Figure 2a shows the XRD patterns of the above four samples. As can be seen from the figure, the diffraction peaks of the GCF samples were located at the same 2θ values as those of the pure copper sheet (JCPDS No.4-836), and there was no impurity peak. The grain size of each sample was approximately 30 nm. XPS analysis (Figure 2b) shows that sample GCF-0.2 was mainly composed of Cu, with Cu_2p3/2_ and Cu_2p1/2_ located at 932.7 and 952.6 eV, respectively, corresponding to Cu^0^ [27], which is consistent with Figure 2a. The weak O1s peak at 530.4 eV indicates that the sample contained a small amount of copper oxide, which may be caused by the oxidation of the surface of copper particles in the air.

**Figure 3:**
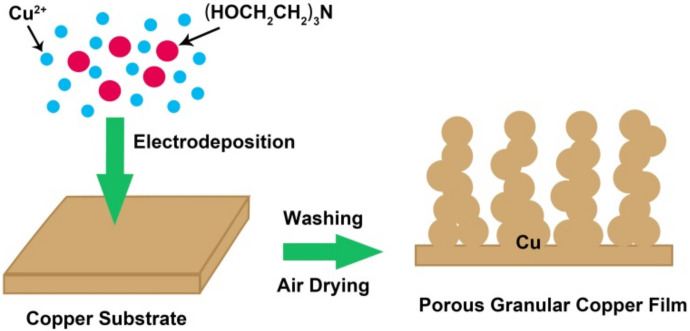
The schematic diagram of the GCF formation process.

Figure 1 indicates that the addition of triethanolamine to the CuSO_4_ solution significantly changed the morphology of the samples. The reason may be ascribed to the fact that triethanolamine molecules can coordinate with copper ions [28], and the large volume and steric effect of triethanolamine molecules would hinder the accumulation of copper ions during the electrodeposition process. Therefore, the electrodeposited metal copper forms a porous granular film structure (Figure 3). The concentration of triethanolamine has an effect on the size of copper particles. As the concentration increases, the particle size of copper particles decreases. However, when the concentration of triethanolamine is too high, the adhesion between the tiny particles of copper weakens, leading to the falling off of copper particles in large areas. According to the experimental results, the optimum additional amount of triethanolamine was 0.2% in weight.

### 3.2. Effect of the Triethanolamine Dosage and pH Values

**Figure 4:**
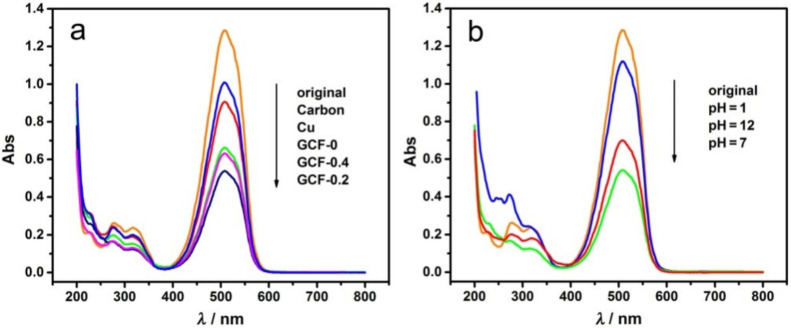
UV–visible absorption spectra of MO before and after decolorization: (**a**) different samples; (**b**) different pH values with GCF-0.2.

Carbon, Cu, GCF-0, GCF-0.2 and GCF-0.4 were used as cathodes, and the other conditions were the same as in Section 2.2 (similarly hereinafter). The UV–visible absorption spectra of MO before and after the reaction are shown in Figure 4a. When the cathode was carbon, Cu, GCF-0, GCF-0.2 or GCF-0.4, the decolorization ratio (DR) of MO was 21.4%, 29.2%, 48.3%, 57.9% or 51.1%, respectively. Evidently, the DR of MO with Cu and GCF cathodes was higher than that with the commonly used carbon electrode. The DR with GCF cathode was much higher than that with the Cu cathode, and the GCF-0.2 cathode exhibited the largest DR of MO.

To study the effects of pH value on the degradation of MO, GCF-0.2 was used as the cathode (similarly hereinafter). MO was degraded at pH = 1, 7 and 12. As shown in Figure 4b, when the pH of MO solution was 1, 7 and 12, the DR was 13.0%, 57.9% and 45.3%. Under neutral conditions, the DR was the highest, which decreased in both alkaline and acidic environments, especially in an acidic environment.

**Figure 5:**
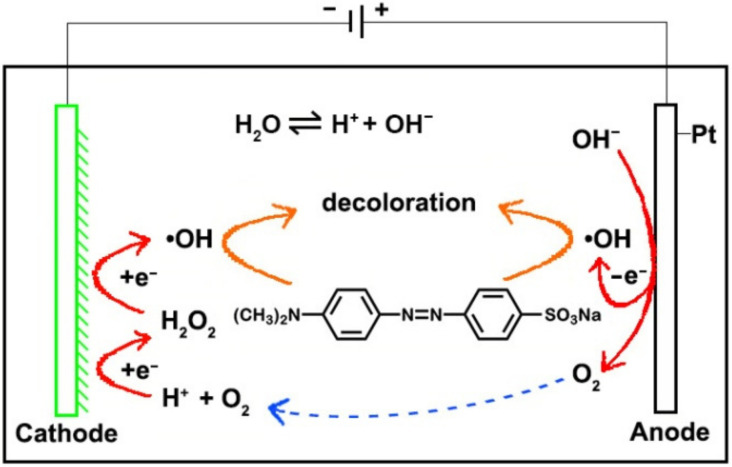
The mechanism diagram of MO decolorization reaction.

It can be seen from the experimental results that the cathode had a great influence on the degradation of MO. As mentioned above, during the electrochemical degradation process, in addition to the oxidation of MO at the anode, the indirect oxidation at the cathode can increase the decolorization rate due to the formation of H_2_O_2_ (Equation (1)). Since Cu and CuO_X_ can act as a Fenton-like catalyst [22,23,24], the indirect oxidation due to electro-Fenton-like reactions at the Cu cathode would accelerate the degradation of MO. This reaction mechanism is shown in Figure 5. The surface of the Cu cathode was smooth and flat, the specific surface area was small and the indirect oxidation was weak. Compared with the original Cu electrode, the GCF samples had a large number of corners, a higher specific surface area and more active sites; thus, the production of H_2_O_2_ and •OH at the GCF cathode increased remarkably. Consequently, a stronger, indirect oxidation would occur at the GCF cathode. As can be seen from Figure 1, the particle size, film thickness and compactness of samples GCF-0, GCF-0.2 and GCF-0.4 were significantly different, as their surface areas were approximately 177.3, 261.5 and 386.2 cm^2^, respectively [29,30]. The sample GCF-0.2 had the largest surface area; therefore, its decolorization rate was the fastest.

Under alkaline conditions, the OH^−^ concentration in solution is high, causing a relatively higher production of O_2_ and •OH on the anode surface (Equations (3) and (4)) [31,32]. The increased amount of •OH is beneficial to MO degradation, but the generated O_2_ would interfere with the oxidation of MO at the anode. Meanwhile, at the cathode, although the amount of available O_2_ is increased, the H^+^ concentration is low, which is not beneficial to the production of H_2_O_2_ and •OH. In other words, multiple factors have led to a reduction in the degradation of MO. Under acidic conditions, the H^+^ concentration in solution is too high, causing a strong hydrogen evolution reaction (Equation (5)), which would greatly inhibit the indirect oxidation of MO at the cathode. At the same time, the too high H^+^ concentration will cause the etching on the cathode. In addition, the too low OH^−^ concentration is not conducive to the oxidation of MO at the anode; consequently, the DR of MO decreases under acidic conditions. Under neutral conditions, the concentrations of OH^−^ and H^+^ are moderate, and the reactions at the anode and cathode are well balanced; thus, the DR of MO is high.

4OH^−^ − 4e^−^ → 2H_2_O + O_2_, (3)

OH^−^ − e^−^ → •OH, (4)

2H^+^ + 2e^−^ → H_2_. (5)

### 3.3. Effect of the Current Intensity

**Figure 6:**
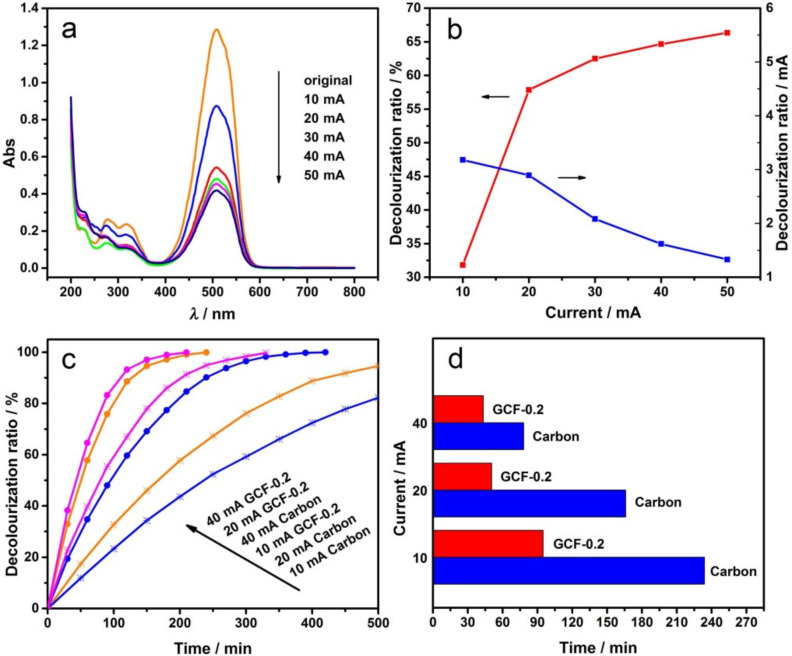
Effect of the current intensity: UV–visible absorption spectra of MO (**a**) and the decolorization ratio curve (**b**) with GCF-0.2 cathode; decolorization ratio-time curve (**c**) and current intensity-time histogram (**d**) when the decolorization ratio is 50% with different cathodes at different current intensities.

The electrochemical degradation experiments were carried out under different current intensities, and the UV–visible absorption spectra are shown in Figure 6a. The DR of MO at different current intensities and the DR curve of unit current intensity (decolorization ratio/mA) are shown in Figure 6b. As the current intensity increased, the DR of MO increased, but the current efficiency decreased in turn. When the current intensity was raised from 10 to 20 mA, the DR significantly improved and the current efficiency slightly lowered. Therefore, further increasing the current intensity would result in the slowly improved DR and remarkably lowered current efficiency. The magnitude of the current intensity reflects the speed of the electrode reactions.

The results in Figure 6a,b suggest that when the current intensity was small, the decolorization rate on the anode and cathode increased along with the increasing current intensities. However, when the current intensity was relatively large, the electrode potential increased, and the side reactions, such as oxygen evolution and hydrogen evolution, were enhanced due to the limited mass transfer rate in the solution. Consequently, the decolorization efficiency of the electrode was lowered. In short, when the current intensity was 20 mA, the system had both a fast decolorization rate and a high power utilization rate; therefore, the best electrochemical degradation current intensity was 20 mA.

MO electrochemical oxidation experiments were carried out with GCF-0.2 cathode and the carbon cathode. The decolorization ratio-time curve under different current intensities is shown in Figure 6c. As can be seen, the DR of MO increased with the increasing current intensities and time; when the DR of MO reached 50%, the decolorization times required for carbon and GCF-0.2 cathodes are shown in Figure 6d. Compared with the commonly used carbon cathode, the decolorization rates of MO with GCF-0.2 cathode increased by approximately 59.4%, 70.9% and 44.6% when the degradation current intensities were 10, 20, and 40 mA. Figure 6c,d indicates that the current intensity had a significant influence on the electro-Fenton-like degradation of MO by GCF-0.2 cathode. The indirect oxidation at the cathode requires O_2_ (Equation (1)), which is mainly generated at the anode. When the current intensity was 10 mA, the O_2_ generated at the anode was less, and the efficiency of the GCF-0.2 cathode cannot be fully exerted. When the current intensity was 40 mA, although more O_2_ generated at the anode, the H_2_ generated at the cathode also increased, which prevents the diffusion of O_2_ to the cathode surface and is not conducive to the cathode reaction. When the current intensity was 20 mA, the effects of both sides can be well balanced. Thus, the GCF-0.2 cathode exhibited a better decolorization effect of MO.

### 3.4. Effect of Degradation Temperatures

**Figure 7:**
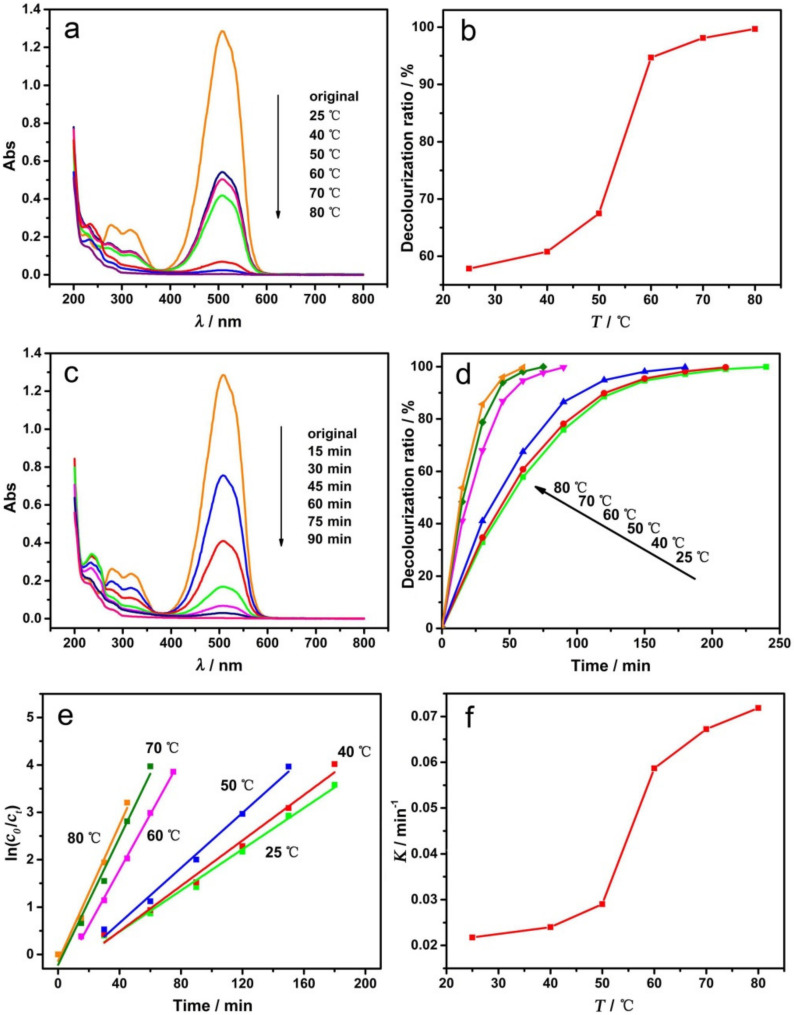
Effect of degradation temperatures with GCF-0.2 cathode: UV–visible absorption spectra of MO at different temperatures (**a**); the decolorization ratio-temperature curve (**b**); UV–visible absorption spectra of MO at 60 °C for different times (**c**); the curve of decolorization ratio versus time at different temperatures (**d**); plots of ln(*c*_0_/*c_t_*) versus time (**e**); curve of the rate constant (K) versus temperature (**f**).

The electrochemical degradation experiments were carried out at different temperatures under a constant current condition. The UV–visible absorption spectra of MO after decolorization are shown in Figure 7a, and the DR of MO at different temperatures are shown in Figure 7b. As indicated, the degradation temperatures can greatly influence the decolorization of MO. The DR increased with the increasing temperature, with a rapid increase appearing at 50–60 °C.

To further explore the effects of temperature on the decolorization reaction, the degradation experiments were carried out by changing the temperature and time. Figure 7c shows the UV–visible absorption spectra of MO at 60 °C for different decolorization times, and Figure 7d shows the curve of DR versus time at different temperatures. It can be seen that, with the prolongation of degradation time, the DR of MO gradually increased until it reached 100% (complete decolorization, the solution is colorless and transparent). When the degradation temperature was 25, 40, 50, 60, or 80 °C, the decolorization rate increased sequentially, and the time required for complete decolorization shortened to approximately 240, 210, 180, 90, 75 and 60 min, respectively.

In order to further investigate the reaction kinetics and the effects of the degradation temperature on the reaction rate, the plots of ln(*c*_0_/*c_t_*) versus time (obtained from Figure 7d) are shown in Figure 7e. The ln(*c*_0_/*c_t_*) is linearly related to time under various temperature conditions, and all linear correlation coefficients (R^2^) are greater than 0.98, indicating that the electrochemical degradation of MO is a first-order reaction. The rate constant (K) of the degradation reaction increased sequentially with temperature and it was 0.02175, 0.02398, 0.02904, 0.05866, 0.06724 and 0.07183 min^−1^ at 25, 40, 50, 60, 70 and 80 °C, respectively. Figure 7f is the corresponding curve of K (rate constant) versus temperature, the shape of which is identical to that of Figure 7b. The rate of electrochemical degradation increased significantly when the reaction temperature was 60 °C or higher.

The main reason that the degradation temperature affects the decolorization ratio is that the increased temperature is able to accelerate the molecular motion speed. Furthermore, at a higher temperature, the structural vibration of the MO molecules would be strengthened, and the activation energy of the reaction would be decreased. Thus, when the temperature is 60 °C or higher, the free radicals with less oxidizability, such as HO_2_• generated on the electrode, can also oxidize the MO molecules, resulting in a dramatic increase in the rate of electrochemical degradation.

**Figure 8:**
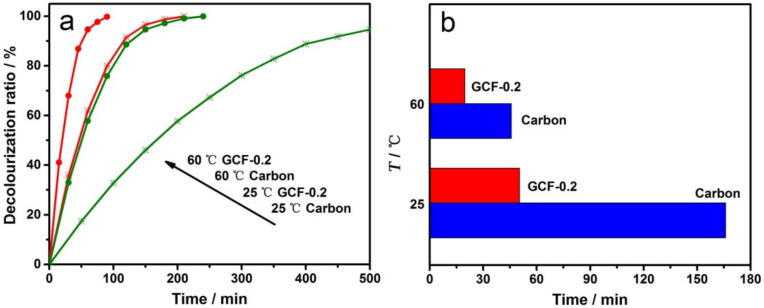
Decolorization ratio-time curves (**a**) and temperature-time histogram when the decolorization ratio is 50% (**b**) with different cathodes at different temperatures.

MO electrochemical degradation experiments were carried out at 25 and 60 °C with the carbon cathode and GCF-0.2 cathode, respectively. The decolorization ratio-time curves are shown in Figure 8a. As can be seen from the figure, when the temperature was raised to 60 °C, the reaction rate was significantly accelerated, regardless of using the carbon or GCF-0.2 cathode. Figure 8b reveals the decolorization times required for the carbon and GCF-0.2 cathodes when the DR of MO reached 50%. At the reaction temperatures of 25 and 60 °C, compared with the carbon cathode, the GCF-0.2 cathode increased the DR of MO by approximately 70.9% and 57.0%, i.e., the elevated temperature did not improve the performance of the GCF-0.2 cathode. As is well known, •OH is a strong oxidant, and the reaction temperature has little effect on its activity. The increasing temperature is more favorable to promoting the direct oxidation at the anode; thereby, the electro-Fenton-like effect of the GCF-0.2 cathode is relatively weakened at 60 °C.

### 3.5. Recycling Tests

**Figure 9:**
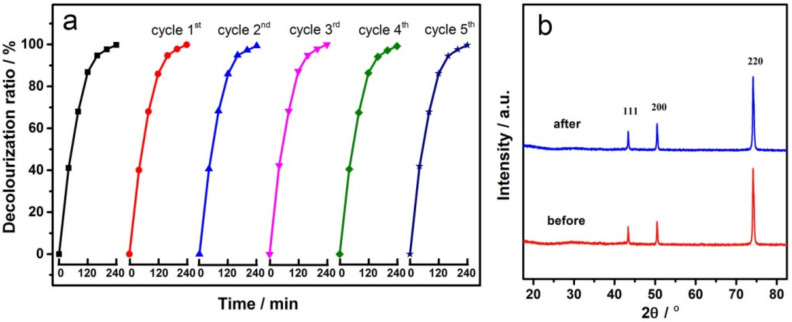
Cyclic performance of GCF-0.2 electrode (**a**) and XRD patterns before and after cycling (**b**).

The stability and reusability of the electrode is very important for practical application. Therefore, the repeatability of the GCF-0.2 sample was tested at 25 °C to degrade MO, and the results are shown in Figure 9a. With the increase in the number of cycles, the performance of the electrode was similar. As can be seen from Figure 9b, the XRD patterns of the electrodes did not change significantly before and after cycling, and both of them only had the diffraction peaks of metal copper. All the above results indicate that the GCF-0.2 electrode has good repeatability and stability.

## 4. Conclusions

In summary, granular copper films (GCFs) were prepared on the surface of copper sheets through the electrodeposition method. Using GCF as a cathode can accelerate the electrochemical degradation of MO. The amount of triethanolamine, pH value, current intensity and degradation temperature have significant effects on the performance of the GCF samples. The morphology and performance of the GCF sample were best when the added amount of triethanolamine was 0.2%. Under neutral conditions, the decolorization rate was largest. Compared with the commonly used carbon cathode, when the temperature was 25 °C and the current intensity was 20 mA (4 mA/cm^2^), and the decolorization rate of MO with GCF-0.2 cathode increased by approximately 70.9%. The electrochemical degradation of MO is a first-order reaction. The decolorization rate increased with the increasing temperature and increased dramatically at 50–60 °C. As well, the GCF cathode had good cycle stability. The significantly improved decolorization rate, neutral reaction solution and good reusability reveal that GCF cathode would have wide application prospects in an electro-Fenton-like system.

## Data Availability

Data available on request. The data presented in this study are available on request from the corresponding author.
